# Hierarchical Porous Graphene Bubbles as Host Materials for Advanced Lithium Sulfur Battery Cathode

**DOI:** 10.3389/fchem.2021.653476

**Published:** 2021-05-24

**Authors:** Wenjie Han, Qing Li, Hua Zhu, Dan Luo, Xianying Qin, Baohua Li

**Affiliations:** ^1^Shenzhen Graphene Innovation Center Co. Ltd., Shenzhen, China; ^2^Tsinghua Shenzhen International Graduate School, Tsinghua University, Shenzhen, China; ^3^Mechanical and Aerospace Engineering Department, University of Missouri, Columbia, MO, United States

**Keywords:** hierarchical pore, graphene bubble, MGO template, lithium sulfur battery, electrochemical performance

## Abstract

The serious shuttle effect, low conductivity, and large volume expansion have been regarded as persistent obstacles for lithium sulfur (Li-S) batteries in its practical application. Carbon materials, such as graphene, are considered as promising cathode hosts to alleviate those critical defects and be possibly coupled with other reinforcement methods to further improve the battery performance. However, the open structure of graphene and the weak interaction with sulfur species restrict its further development for hosting sulfur. Herein, a rational geometrical design of hierarchical porous graphene-like bubbles (PGBs) as a cathode host of the Li-S system was prepared by employing magnesium oxide (MgO) nanoparticles as templates for carbonization, potassium hydroxide (KOH) as activation agent, and car tal pitch as a carbon source. The synthesized PGBs owns a very thin carbon layer around 5 nm that can be comparable to graphite nanosheets. Its high content of mesoporous and interconnected curved structure can effectively entrap sulfur species and impose restrictions on their diffusion and shuttle effect, leading to a much stable electrochemical performance. The reversible capacity of PGBs@S 0.3 C still can be maintained at 831 mAh g^−1^ after 100 cycles and 512 mAh g^−1^ after 500 cycles.

## Introduction

The rapid development of portable electronics and automobiles has brought about new opportunities and challenges to energy storage strategies. Considering the limited energy density of the systems less than 200 Wh/kg (Armand and Tarascon, 2008; Bruce et al., 2011; Goodenough and Manthiram, 2014), the existing lithium ion batteries are still not enough to satisfy many applications. Lots of work have been done to improve the commercial battery system for higher capacity and more stable electrochemistry performance, whereas their energy density is still astricted by its indigenous theoretical capacity (Choi et al., 2012; Wu et al., 2015). Abundant reserves in nature, sulfur attracts extensive attention for superior electrochemical capacity (1,675 mAh g^−1^) and high theoretical energy density (2,600 Wh/kg) (Sun et al., 2013; Xu et al., 2020). However, the insulation nature of active sulfur materials, the severe shuttle effect of the soluble intermediate (Li_2_S_x_, 8 ≥ x ≥ 3), and the expansion and shrinking (around 80%) during the charge/discharge process actually will cause some fatal problems, including low utilization coefficient of sulfur and inferior Columbic efficiency (CE) accompanied with rapid capacity fading, which definitively hampers the practical applications of lithium sulfur (Li-S) batteries (Seh et al., 2013; Wei Seh et al., 2013; Li et al., 2016; Xu et al., 2020). To achieve an advanced Li-S system with stable electrochemical performances, extensive research efforts have been exploited in enhancing the integrated conductivity of cathode and developing an efficient host to confine polysulfides (Yin et al., 2012; Liu et al., 2017; Tao et al., 2018). Researchers have proposed significant strategies to improve the conductivity in cathode by introducing highly conductive carbon matrix (e.g. porous carbon sphere, carbon nanotube, graphene, etc.) or conductive polymers (i.e., polypyrrole, polyaniline, polythiophene, etc.) to host the insulate sulfur (Ji et al., 2009; Sun et al., 2012; Li G. et al., 2015). Among diversified approaches to address the obstacles of the Li-S system, integrating sulfur into porous carbon matrix is one of the most common ways both having convenience and effectiveness (Seh et al., 2013; Huang et al., 2014). Porous carbon matrix can provide sufficient electroactive sites for electrochemical reactions and highly efficient transfer paths for irons and electrons. The porous structure, large surface area, strong adsorption ability, and superior conductivity are contributed to facilitating carbon host materials to confine the shuttle effect of polysulfides (He et al., 2014; Zhang et al., 2016).

Graphene is a favorable carbon host due to its superior specific surface area and excellent electrical conductivity (Peng et al., 2014; Wu et al., 2017). Nevertheless, the non-defective two-dimensional (2D) structure will show deadly shortcomings where polysulfides may easily leak out of the cathode part and ions are not able to pass through the barrier-like sheets swimmingly (Su et al., 2012; Zhang et al., 2019). Thus, functionalized and holey graphene nanosheets are developed to immobilize polysulfides and facilitate ion transport (Huang et al., 2016; Chang et al., 2017; Du et al., 2019). Furthermore, three-dimensional (3D) graphene framework is designed and fabricated as an effective host material for Li-S batteries due to its hierarchically porous structure and interconnected carbon scaffold for electron and ion transport, as well as polysulfide storage and immobilization. In the hierarchical structure, macrospores can supply kinetically favorable mass transportation channels and buffer volume expansion during lithium insertion/extraction process, and mesopores and micropores are able to trap polysulfides efficiently, so as to prevent from the inherent diffusion and shuttling effect (Lee et al., 2017; Zhang et al., 2019). Recently, crumpled graphene microflower is developed as an effective host material for Li-S batteries on the basis of the spray-drying technique using ultra large graphene oxide as a raw material (Chen et al., 2017). Besides graphene-based porous host materials, interconnected porous bubble-like carbon also can be achieved by regarding other low-cost but effective resources as carbon precursors, such as sucrose (Zhang et al., 2014), resin (Zheng et al., 2019), pitch (He et al., 2014), and so on. Zhang and co-workers fabricated interconnected porous carbon bubbles with a hollow space of 70 nm and a carbon shell of 12 nm, which are utilized as host materials to accommodate selenium (Se) for high-performance Li-Se batteries (Zhang et al., 2014). Li et al. reported a rational design and synthesis of sulfur/carbon (S/C) nanocomposites by infiltrating into 3D graphene-like material with hierarchical pores, which was originated from ion-exchange resin (Li et al., 2014).

Pitch is also a paramount precursor for the high-conductive carbon material (Qin et al., 2012). Mesoporous carbon hollow spheres derived from petroleum pitch based on a silica template approach are applied to encapsulate and sequester elemental sulfur in their interior and porous shell (Jayaprakash et al., 2011). On the other hand, the large internal void space, cracked carbon shell, and monodispersed structure of the hollow carbon result in a low Coulombic efficiency (CE) of <95% for the S/C cathode during the cycle process. He and co-authors reported hollow porous graphene balls (HPGBs) directly synthesized from coal tar pitch by a simple hard template strategy coupled with activation (He et al., 2014). As electrode materials for supercapacitors, the HPGBs featured by a 3D interconnected architecture reveal a high specific capacitance, an excellent rate performance, and a good cycle stability on account of its hierarchical porous structure and high specific surface area.

Herein, the synthesis of 3D porous graphene bubbles (PGBs) that have an interconnected structure and hierarchical pores via a one-step template-assistant carbonization–activation method is put forward. In this system, pitch-coated magnesium oxide (MgO) is converted into hierarchical porous graphene-coated MgO during the carbonization and activation process. Subsequently, the byproducts of potassium hydroxide (KOH) and MgO template are removed by acid treatment. MgO is used as a sacrificial template to create inner void space in the final product and guarantee the formation of graphene-like thin carbon layer among these NPs. As host materials for Li-S battery cathode, the resultant 3D PGBs have several merits: (i) the architecture with hierarchical porous external shell and internal void (100 nm) can accommodate large amount of sulfur and effectively mitigate its volume expansion; (ii) the thin shell and 3D framework structure of PGBs can provide efficient pathways for the electronic transfer between active materials and current collectors; and (iii) the high porosity of graphene shell and the 3D framework structure of PGBs can enhance the electrolyte penetration during the electrochemical test and significantly enhance the ionic diffusion efficiency between electrolytes and active materials by enlarging the contact area and reducing the diffusion pathways.

## Experimental Design

### Material Preparation

#### Preparation of PGBs

Nine g MgO nanoparticles (50 nm), 6 g KOH from Aladdin, and 2.5 g coal tar pitch (softening point around 200°C from Donghua University) were uniformly ground in N,N-dimethylformamide (DMF) to form a slurry-like mixture, which was dried at 60°C overnight. Then, the mixed powder was put into a corundum crucible and heated at a 5°C/min rate to 220°C for 2 h to soften the coal tar pitch and was further carbonized at 850°C for 3 h with the same heating rate. After cooling down to room temperature naturally, the sample was washed with superfluous 1 M hydrochloric acid (HCl) at 60°C to completely remove the MgO template, followed by vacuum filtrating and washing with distilled water. The PGBs were collected after thorough drying at 80°C for overnight.

Commercial graphene powder was directly bought from Garmery Graphene Marketing Center without further treatment.

#### Preparation of PGBs@S and G@S Composite

To obtain PGBs@sulfur (PGBs@S) and graphene@sulfur (G@S) composites, 60 wt.% sulfur and 40 wt.% PGBs or graphene powder was uniformly ground and then transferred to a sealed vessel filled with N_2_ protection, which was heated at 155°C for 12 h to infiltrate sulfur, followed by additional heating at 200°C for 0.5 h to vaporize the superfluous sulfur on the outer surface of the PGBs@S and G@S hybrids.

### Characterization of PGBs and Graphene Powder

The morphologies and structure of PGBs were observed by field emission scanning electron microscope (SEM, Zeiss SUPRA 55) and a high-resolution transmission electron microscope (TEM, FEI TECNAIG2 F30). Nitrogen adsorption isotherms and Brunauer–Emmett–Teller (BET) surface area were measured with Micromeritics ASAP 2020. The sulfur weight content in the CNF-T interlayer was measured by thermogravimetric analysis (TGA, NETZSCH STA449F3). The Raman spectra were obtained *via* a Raman spectroscopy (HORIBA LabRAM HR Evolution) with a 532 nm Ar-ion laser.

### Electrochemical Characterization

The electrochemical measurements were carried out with 2,032 coin cells by applying 80 wt.% PGBs@S or G@S, 10 wt.% carbon black (Super P), and 10 wt.% polyvinylidene fluoride (PVDF) dissolved in N-methyl pyrrolidone (NMP). The slurry was coated on a carbon-coated aluminum foil using a doctor blade and dried at 60°C for 24 h. The mass loading of sulfur of the obtained composite cathode was ~1.2 mg cm^−2^ for normal electrodes and ~4.2 mg cm^−2^ for the high loading ones. The electrolyte was 1 M bis(trifluoromethane)sulfonimide lithium salt (LiTFSI) dissolved in a mixture of 1,2-dioxolane (DOL) and dimethoxymethane (DME) (1:1 by volume) with 1 wt.% LiNO_3_. The amount of electrolyte in the cells is 60 μl. A Celgard 2400 membrane was chosen as the separator. The cathode electrodes were cut into disks of 12 mm in diameters for cell assembling. The coin cells were cycled between 1.7 and 2.8 V on a LAND 2001 CT battery tester at ambient temperature. Cyclic voltammetry (CV) experiments within the voltage range of 1.7–2.8 V at a scan rate of 0.1 mv s^−1^ and electrochemical impedance spectroscopy (EIS) measurement obtained at a constant perturbation amplitude of 5 mV in the frequency range between 0.01 and 100 kHz were carried out using a Solartron electrochemical workstation (Bio-Logic Science Instruments).

## Results and Discussion

**Figure 1:**
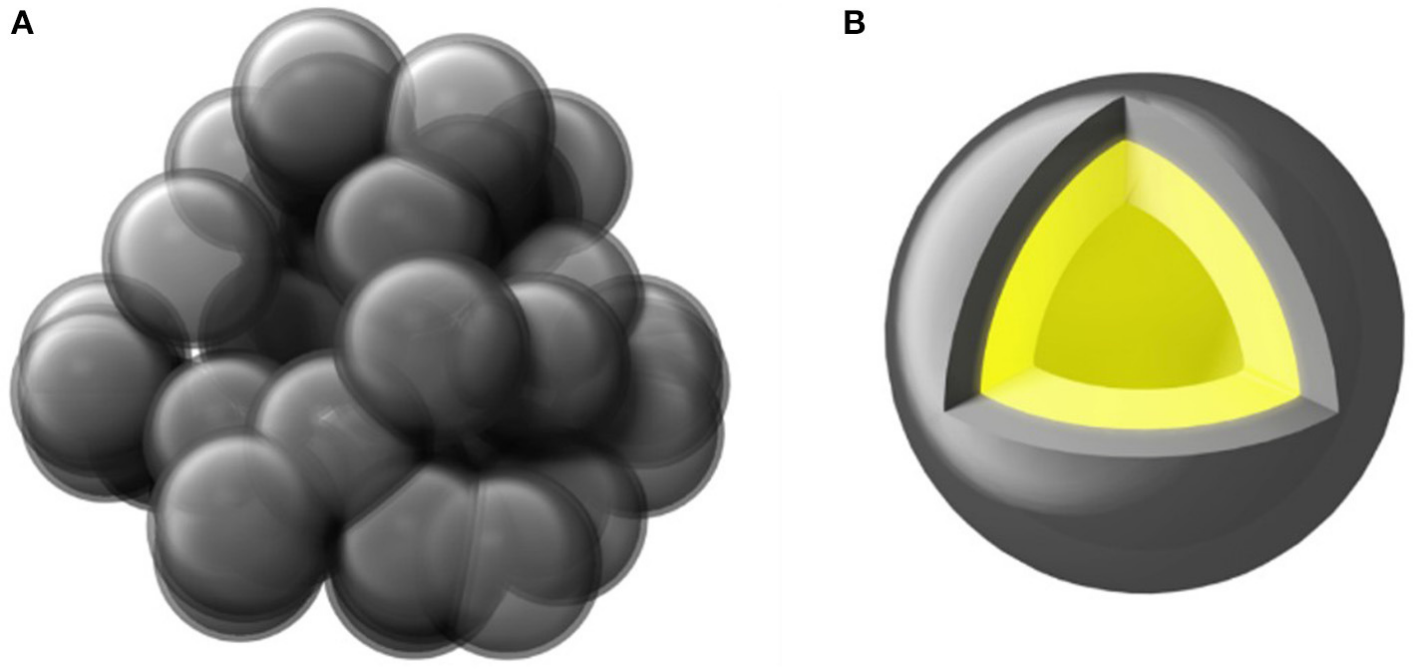
The schematic diagrams of **(A)** PGBs and **(B)** PGBs@S.

The schematic diagram of the synthesized PGBs is shown in Figure 1A. The sphere-like bubbles are left by etching MgO templates from the carbon matrix, and the carbon source of coal tar pitch with a superior flowability above the soft-point temperature contributes to the interconnected porous carbon framework, instead of a dispersed carbon sphere (Zhang et al., 2015). Meanwhile, micro- and mesopores are created by KOH activation and well-distributed in the carbon layer. When sulfur is infiltrated into the PGBs, it is supposed to be uniformly distributed in the carbon layer and encapsulated in the internal space of PGBs, which is shown in Figure 1B. The porous structure and the connected carbon layer will supply the infiltrated and encapsulated sulfur with the double protection, preventing the resultant polysulfides from diffusion and shuttling.

**Figure 2:**
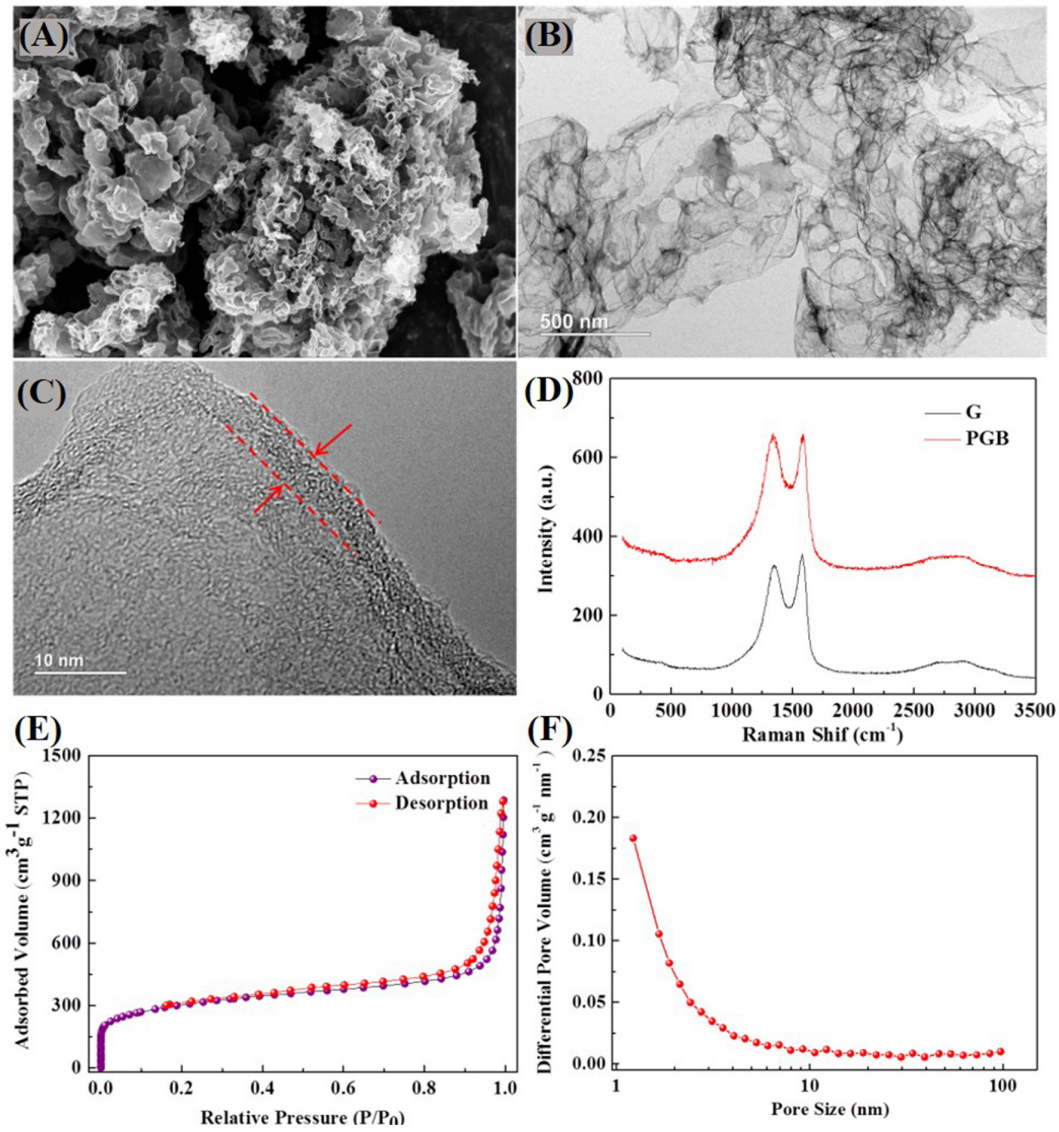
**(A)** SEM, **(B)** TEM, and **(C)** HRTEM images of the synthesized PGBs; **(D)** Raman spectra of PGBs and obtained graphene powder, **(E)** nitrogen adsorption–desorption isotherms, and **(F)** pore size distribution of PGBs.

The morphology of PGBs is characterized by SEM and TEM images as illustrated in Figures 2A,B, respectively. The PGBs reveal a well-connected and porous structure under SEM observation that is conductive to cultivate the persistent conductivity and unexceptionable capture capacity for polysulfides in Li-S batteries. The detailed structure can be further observed from the TEM images in Figure 2B, showing that macropores of 50–100 nm are generated by the MgO template in the carbon skeleton. It is worth noting that the PGBs possess an ultrathin shell about 5 nm, which is present in Figure 2C. By contrast, the SEM images of obtained graphene powder are shown in Figure 1B and Supplementary Figure 1A, and the carbon structure rather plated with open gaps between layers cannot effectively entrap the polysulfides, causing a fast capacity decay of the cells. As shown in Supplementary Figure 2, the SEM image of G@S demonstrated an accumulated structure with no sign of initial layered structures, suggesting that the sulfur is on the outer surface and has not been well-incorporated. Raman spectra for commercial graphene and the synthesized PGBs are conducted to further elaborate the carbon structure, shown in Figure 2D. The typical peaks of D band at around 1,320 cm^−1^, of G band at around 1,590 cm^−1^, and of 2D bands at around 2,700 cm^−1^ are obviously observed for both PGBs and commercial graphene powder (Malard et al., 2009). Through calculation, the intensity ratio IG/ID of PGBs is 1.01, which is very close to that of graphene (1.07). Meanwhile, the intensity of 2D peak for PGBs is also similar to that of graphene powder. The high IG/ID and obvious 2D peak indicate the high graphitization degree of the PGBs. The adsorption–desorption curve shows a typical type IV isotherms plot, and the obvious hysteresis loop reveals a typical presence of micro- and mesopores in Figure 2E and Supplementary Figure 3A. It shows that PGBs have a higher BET surface area of 1,073.6 m^2^ g^−1^ and a pore volume of 1.40 cm^3^ g^−1^, whereas the obtained graphene powder has a lower surface area of 607.7 m^2^ g^−1^ and a high pore volume of 4.85 cm^3^ g^−1^. The BJH pore size distribution of both PGBs and graphene powder in Figure 2F and Supplementary Figure 3 shows the hierarchical pore size distribution with wide peaks in the area of micro- and mesopores. The BET mean pore diameter of PGBs is 5.20 nm, which is much smaller than the value of 31.95 nm for graphene powder. The large number of pores and suitable pore structure can further validate that the sulfur has been well-incorporated into the carbon skeleton. The BET surface area of PGBs@S is 20.35 m^2^ g^−1^ and pore volume of 0.01 cm^3^ g^−1^, indicating that sulfur has been well-incorporated. The higher BET surface and narrower pore width for PGBs indicate the abundant micro- and mesopores inside, which can limit the polysulfides leakage. Thanks to the interconnected and curved structure, the suitable pore volume of PGBs is able to offer enough space to sulfur loading and double protection from shuttling. In contrast, the graphene powder owns a larger pore volume and mean pore width but a smaller BET surface area, which can validate its open structure and larger pores, creating the fast polysulfide shuttling process.

**Figure 3:**
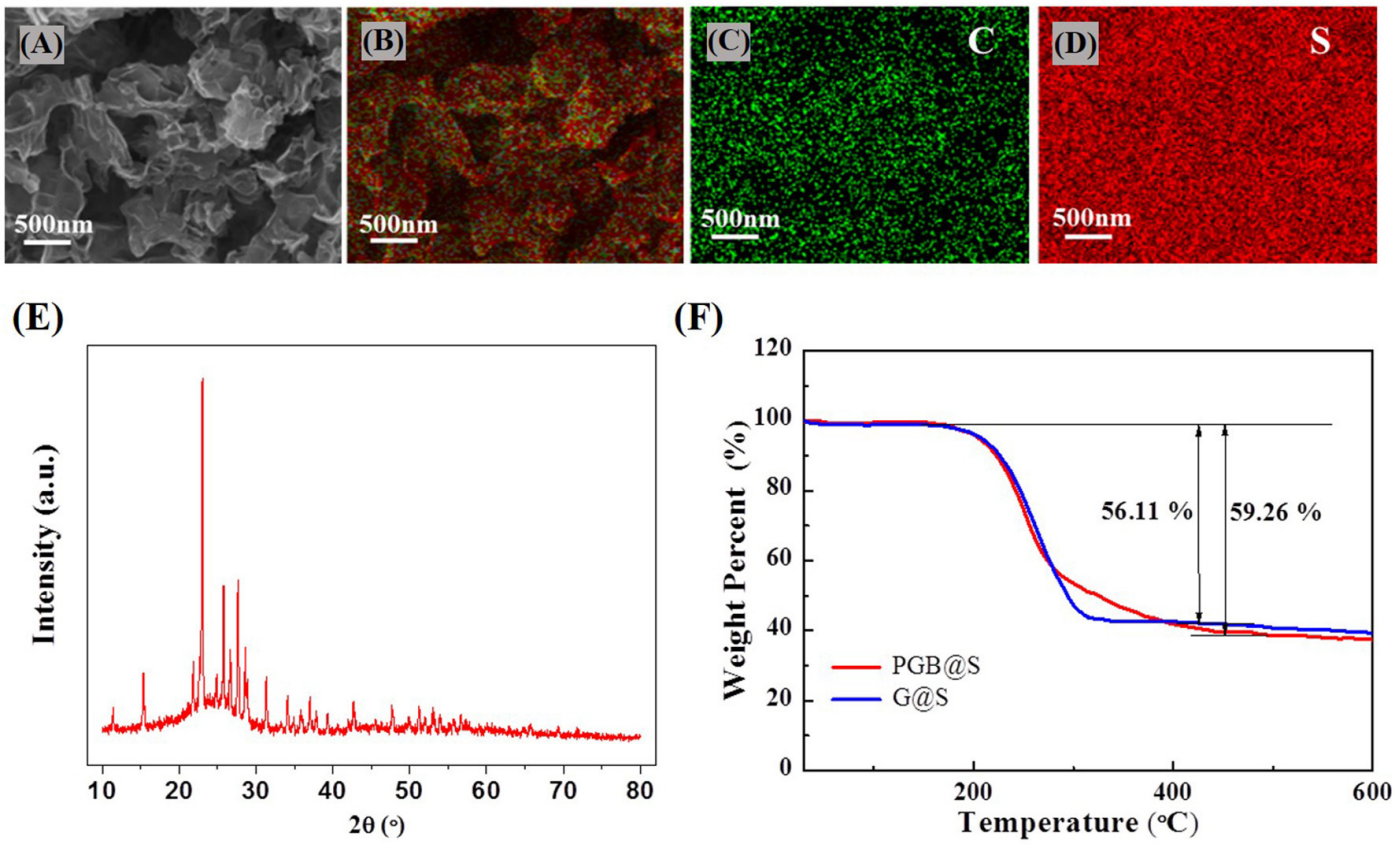
**(A)** SEM images and **(B)** corresponding element mapping of PGBs@S, **(C)** carbon and **(D)** sulfur; **(E)** XRD spectra of PGBs@S; **(F)** TGA analysis of PGBs@S and G@S.

The X-ray diffraction (XRD) spectra of the PGBs have demonstrated obvious carbon peaks, and there is no MgO peak in the XRD, which indicates that MgO was completely removed, as shown in Supplementary Figure 4. After infiltrating sulfur into PGBs by heat treatment, the sulfur is uniformly distributed in the porous carbon matrix from the EDS analysis results, as illustrated in Figures 3A–D. The XRD spectra for the PGBs@S in Figure 3E also reveal both distinct sulfur peaks and carbon peaks indicating a uniform mixture of the two elements. To identify the sulfur content in the composites, TGA that is implemented is present in Figure 3F. The sulfur ratios in PGBs@S and G@S are 59.26 and 56.11%, respectively, implying that the weight loss of the excrescent sulfur in PGBs@S is much less than that in G@S, indicating that sulfur in PGBs@S is well-infiltrated inside the carbon matrix. Evidently, the temperature for complete sulfur loss in PGBs@S is seen at around 400°C, much higher than that in G@S at around 300°C. The higher temperature range for complete weight loss of sulfur in PGBs@S notes the better incorporation of sulfur in PGBs matrix, which may result from the unique bubble-like clusters with enclosed but interconnected structure, turning out that the weight loss process of sulfur in PGBs is more difficult than graphene host with an open structure.

**Figure 4:**
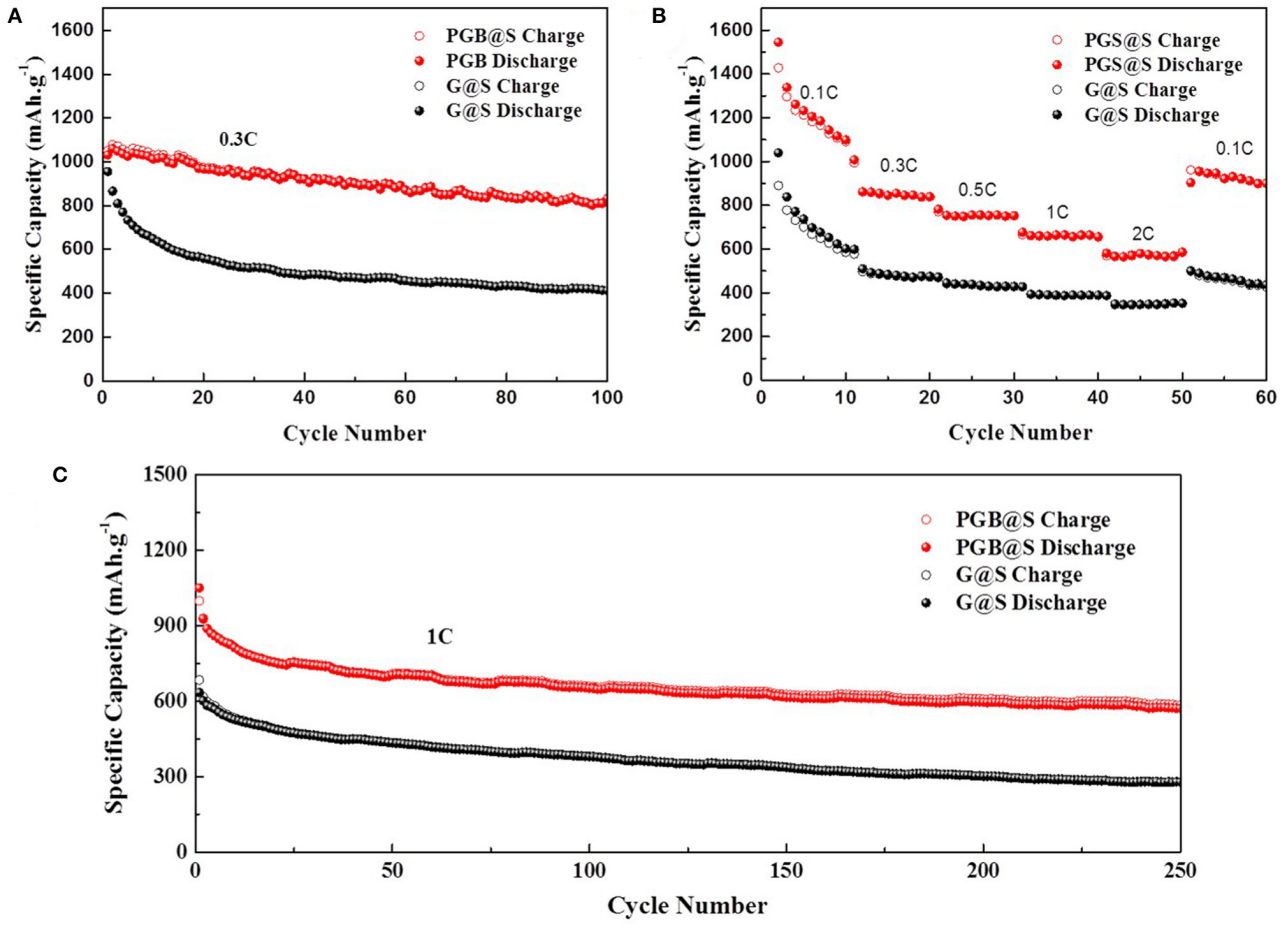
**(A)** Cycling performances at 0.3 C, **(B)** rate capacities, and **(C)** cycling performance at 1 C of PGBs@S and G@S cells.

**Figure 5:**
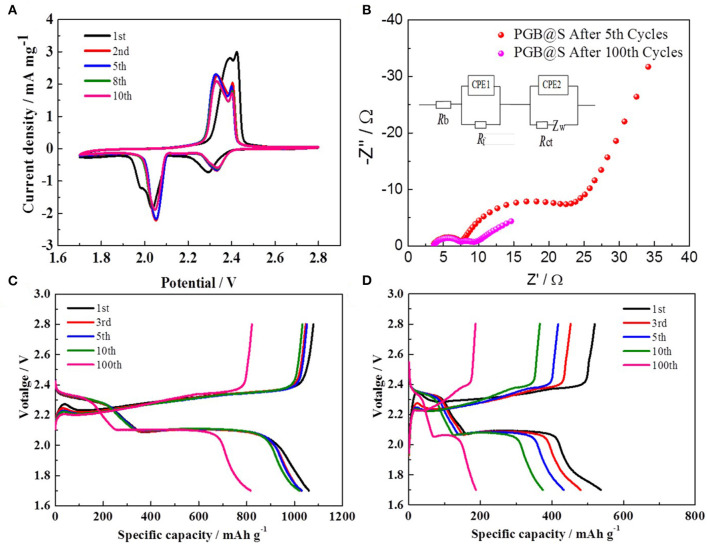
**(A)** CV curves of PGBs@S cell; **(B)** Nyquist plots after 5th cycles from 10 mHZ to 100 kHZ; discharge–charge curves of **(C)** PGBs@S and **(D)** G@S batteries.

The electrochemical performances of PGBs@S and G@S are characterized by assembling the cells with Li foil as an anode, and the test results are shown in Figures 4, 5. A 0.3 C current density was applied to evaluate the cycling performance of PGBs@S and G@S in Figure 4A, and the initial capacity of PGBs@S is about 1,031.1 mAh g^−1^ which is slightly higher than G@S with 955.3 mAh g^−1^. In the following 10 cycles, G@S suffers from a fast capacity decay at 649 mAh g^−1^, whereas PGBs@S is well-sustained at 1,030.6 mAh g^−1^. It is well-known that the initial 10 cycles is an essential stage to analyze the leakage of polysulfides from cathode. The reason for this phenomenon may be that the hierarchical bubble structure of PGBs can effectively restrain the active material from losing; in contrast, the polysulfide can easily release the trap of the open structure of graphene, which can be observed from Supplementary Figure 1. The capacity of the PGBs@S cell for the entire remaining 90 cycles at 0.3 C is all higher than that of G@S, and the reversible capacity after 100 cycles can still be preserved at 831 mAh g^−1^. To manifest the long-term cycling stability, the PGBs@S battery is cycled lasting to 500 cycles at 0.3 C. From the results in Supplementary Figure 5, a high capacity of over 500 mAh g^−1^ is observed after 500 cycles, demonstrating the long-term stability at the small current density. As shown in Supplementary Figure 6, when the sulfur loading increased to about 4.2 mg cm^−2^, the PGBs@S battery still has an initial reversible specific capacity at 765.5 mAh g^−1^ and an initial CE of 95.8% at 0.1 C. After 100 cycles at 0.3 C, the reversible specific capacity at 490.5 mAh g^−1^ was still maintained. The rate capacities of PGBs@S and G@S are evaluated in the following current densities: 0.1, 0.3, 0.5, 1, and 2 C noted in Figure 4B. The capacity of PGBs@S is much higher than that of G@S in the whole rate test process. Notably, the capacity of PGBs@S is about 600 mAh g^−1^ in the high current density of 2 C, whereas that of G@S drops to <400 mAh g^−1^ on equal conditions. What is more, when the current density is recovered to 0.1 C, the PGBs@S can return to the high capacity about 1,000 mAh g^−1^, which manifests the higher reversibility. With the purpose of further identifying the cycling ability of PGBs@S in high rate, the cycling experiment in 1 C is conducted, and the results can be observed from Figure 4C. The initial capacities of PGBs@S and G@S are 999.6 and 634.6 mAh g^−1^, respectively. By comparing the initial capacity at 0.3 C, PGBs@S at 1 C is analogical, but G@S shows a sharp drop from 955.3 (0.3 C) to 634.6 mAh g^−1^ (1 C). This is another evidence of the high conductivity, protective structure of PGBs, and a high sulfur utilization rate in PGBs@S. Obviously, it can be observed from Figure 4C that PGBs@S also has a high stability at 1 C with 583 mAh g^−1^ after 250 cycles, which is more than twice of the retained capacity of G@S.

Figure 5A displays the typical CV curves at a scan rate of 0.1 mV s^−1^ for the PGBs@S cell. Two main oxidation peaks are obviously observed at 2.32 and 2.39 V, except for the initial cycles that may be additional polarization for the volume expansion of sulfur (Ye et al., 2015). Correspondingly, those two peaks are equivalent to the transformation process of lithium sulfides to the high-order polysulfides, and further convert to elemental sulfur. The two reduction peaks at 2.32 and 2.05 V can be allocated to the reversal process for reaction to happen in the oxidation peaks, which are highly consistent with the literature reported (Li W. et al., 2015). What is worth mentioning is that the curves are well-overlapped with each other from the 2nd cycle to the 10th cycle, showing the good reversibility of the cell and low polarization as cycling.

The plateaus of galvanostatic charge/discharge profiles in Figures 5C,D are all corresponding to the cathodic and anodic peaks in CV curves. For the PGBs@S cell, the profiles within 10 cycles represented in Figure 5C are all in good consistency, whereas those profiles of the G@S cell exhibit the capacity of gradual decrease for both two discharge plateaus and charge curves. From the profiles of PGBs@S and G@S in the 100th cycle, it is easy to demonstrate the high polarization within the cycling process of G@S which may be attributed to the loss of active materials, whereas the PGBs@S cell displays a rather slow decay, stable plateaus, and higher capacity retention. Figure 5B reveals the EIS plots of the PGBs@S cells after 5 and 100 cycles of charge and discharge. The EIS spectra are composed of two semi-circles and a slope line, which refer to charge transfer resistance (R_ct_), interface contact impedance (R_sei_), and the Warburg impedance (Z_w_), respectively. With the battery cycling, R_ct_ remains at similar value, but R_sei_ is decreased after 100 cycles, which may be caused by the activation process (Yao et al., 2018; Kim et al., 2019). Another reason that the conductivity of cathode is increased is due to the dissolution of polysulfides from the cathode (Ansari et al., 2019; Liu et al., 2019).

**Figure 6:**
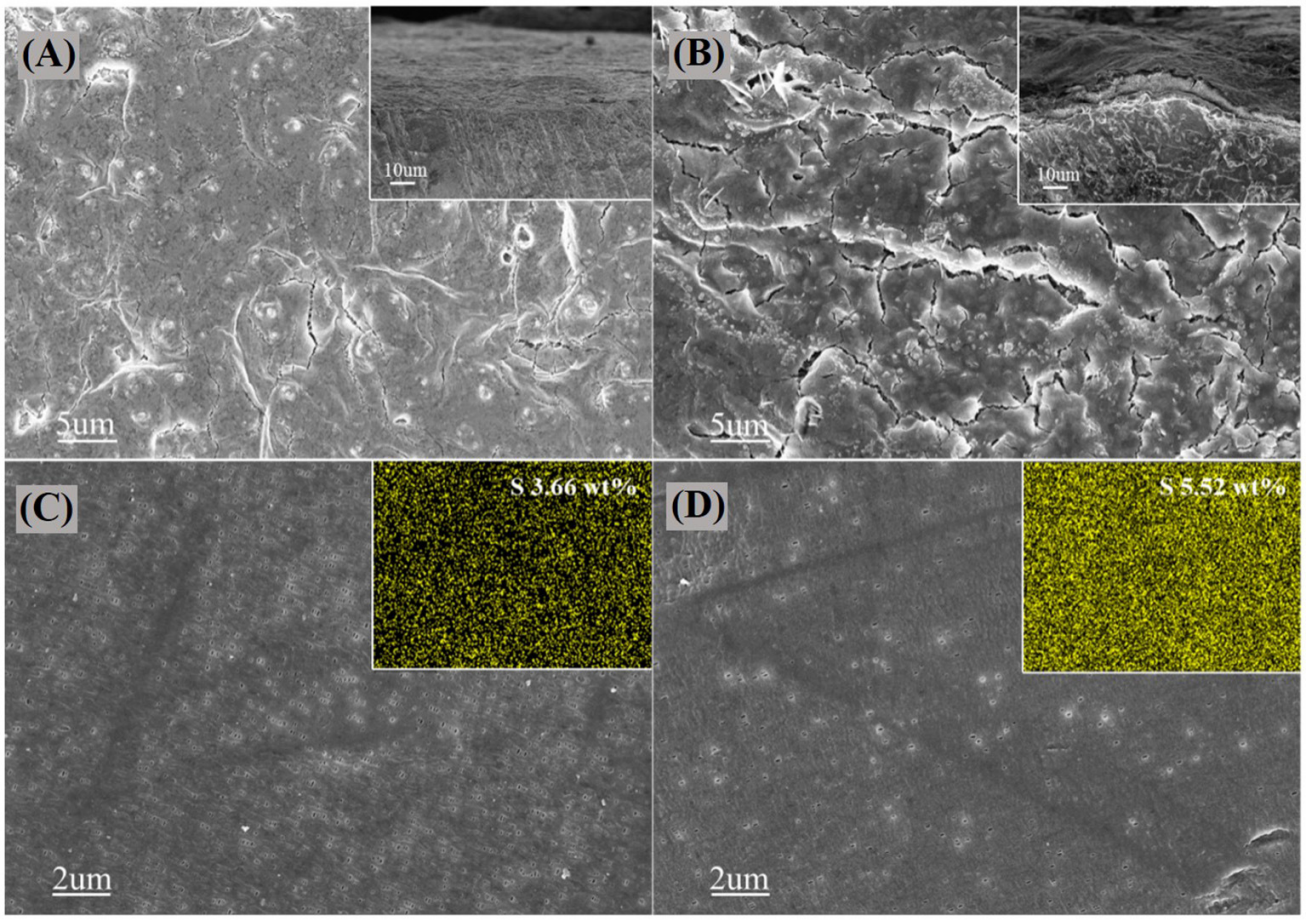
SEM images on the surface and cross-section of cycled lithium foil of **(A)** PGBs@S and **(B)** G@S batteris; SEM images and EDS mapping of the separator surface facing lithium foil side in **(C)** PGBs@S and **(D)** G@S batteries.

It is well-known that the shuttle effect of polysulfides leads to the deposition of Li_2_S and Li_2_S_2_ on the surface of lithium anodes, causing the irreversibility and capacity fading. In order to further comprehend the effect of PGBs host caused by the cathode shuttling and lithium anode in the Li-S system, SEM is conducted for the surface and cross-section of the lithium anode of the PGBs@S and G@S cells after 60 cycles at 1 C rate, respectively. As shown in Figure 6A, the surface of the lithium foil from the PGBs@S cell is rather flat without obvious indication of the growth of lithium dendrite and the deposition of lithium sulfides. The lithium anode from the G@S cell proven in Figure 6B presents a rough waved surface, and an extra layer is clearly witnessed in the cross-section figure, which may arise from the side reaction between polysulfides and lithium accompanied by shuttle effect. The SEM characterization is carried out for the separator facing the lithium anode side in Figures 6C,D to further validate the deduction. It is observed that the pores in the separator from the PGBs@S cell can maintain the initial state, whereas the pores are mostly blocked in the separator of the G@S cell. The EDS mapping of the separator also supports the hypothesis, and the average weight content of sulfur species in the separator for the PGBs@S battery is about 3.66%, which is much lower than that for G@S separator (5.52%), proving the smaller shuttled sulfur amount for the designed PGBs sulfur host.

## Conclusions

In summary, the PGBs with a hierarchical structure were synthesized by using the template-assistant one-step carbonization–activation process, which was further utilized as a host material to incorporate with sulfur for high-performance cathode of Li-S batteries. The synthesized 3D PGBs consist of interconnected hollow carbon bubbles with abundant mesopores, which can encapsulate much sulfur and confine its dissolution into electrolyte. Furthermore, the enclosed framework of PGBs constructed by the curved and interconnected graphene-like nanosheets can further prevent the polysulfides from further shuttling. Therefore, the PGBs can be used as an effective sulfur host for Li-S batteries with the optimal electrochemical performances in this work. The superior electrochemical stability of PGBs that is better than the commercial graphene endows the PGBs with the promising potential to be further developed to multifunctional sulfur host combining with other functional groups or compounds to achieve even better electrochemical performances in the subsequent study.

## Data Availability Statement

The original contributions presented in the study are included in the article/Supplementary Material, further inquiries can be directed to the corresponding author.

## Author Contributions

WH: investigation and writing—original draft. QL, HZ, DL, XQ, and BL: writing—review and editing. XQ: supervision. All authors contributed to the article and approved the submitted version.

## Conflict of Interest

XQ, WH, and DL were employed Shenzhen Graphene Innovation Center Co. Ltd., Shenzhen, China. The remaining authors declare that the research was conducted in the absence of any commercial or financial relationships that could be construed as a potential conflict of interest.
