# Supplementary material for: Hierarchical Porous Graphene Bubbles as Host Materials for Advanced Lithium Sulfur Battery Cathode
Source: Front Chem. 2021 May 24;9:653476. doi: 10.3389/fchem.2021.653476 (PMC8181144; doi:10.3389/fchem.2021.653476)
Supplement: Supplementary file 1 [file Data_Sheet_1.pdf]

Supplementary Material

Hierarchical porous graphene bubble as host materials for advanced lithium sulfur battery cathode

Wenjie Han [1,2], Qing Li [2], Hua Zhu [3], Dan Luo [1], Xianying Qin [1,2*], Baohua Li [2]

[1]Shenzhen Graphene Innovation Center Co. Ltd., Shenzhen, China.

[2]Tsinghua Shenzhen International Graduate School, Tsinghua University, Shenzhen, China.

[3]Mechanical and Aerospace Engineering Department, University of Missouri–Columbia, Columbia, USA.

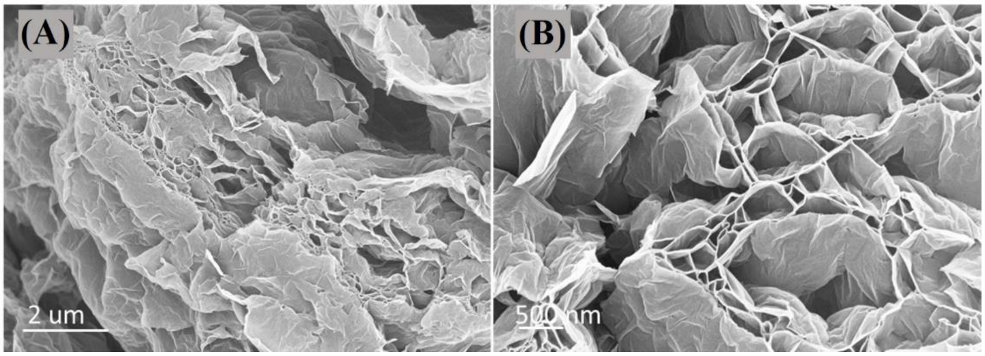

Figure S1 The SEM images of commercial graphene powder.

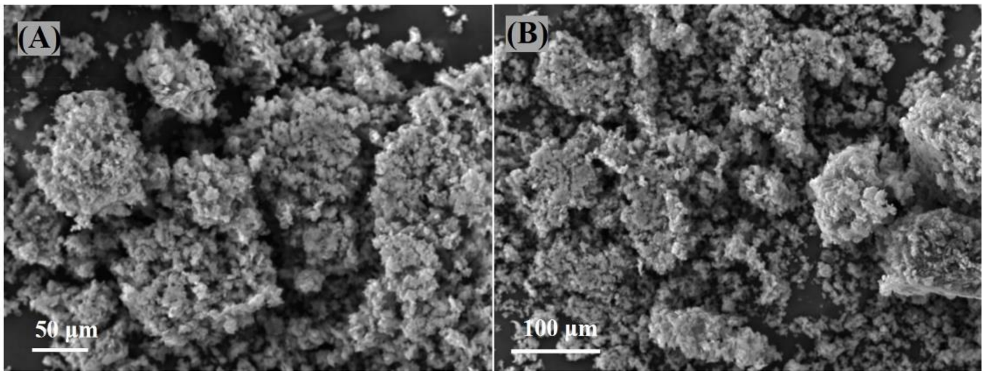

Figure S2 The SEM images of G@S composite.

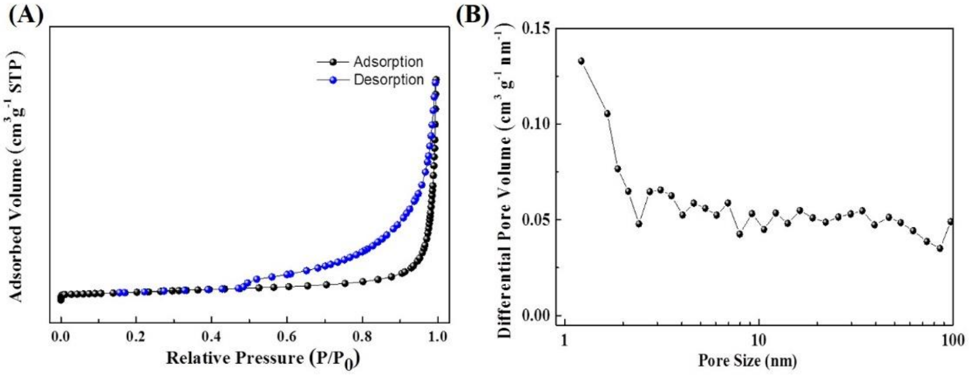

Figure S3 The Nitrogen adsorption–desorption isotherms (A) and pore size distribution (B) of commercial graphene powder.

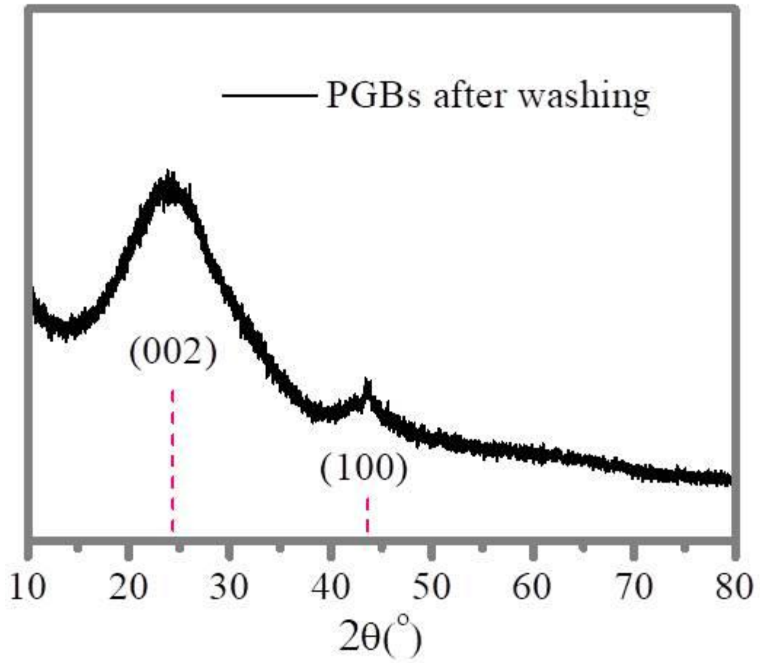

Figure S4 The XRD pattern of PGBs.

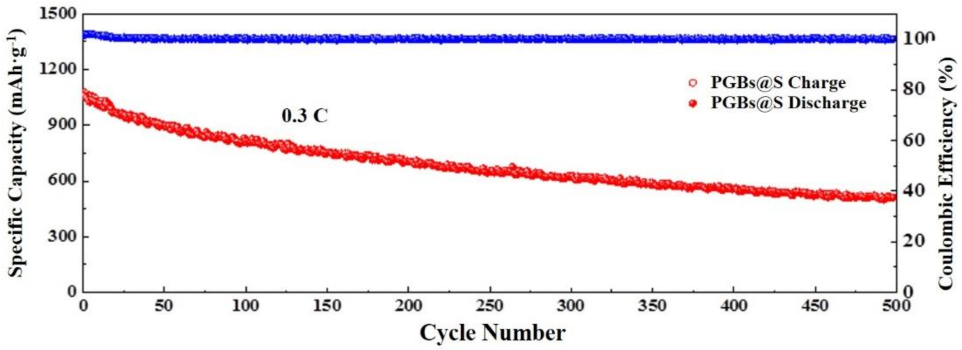

Figure S5 The long cycle performance of PGBs@S at 0.3 C with a sulfur loading of 1.2 g cm^{-2}.

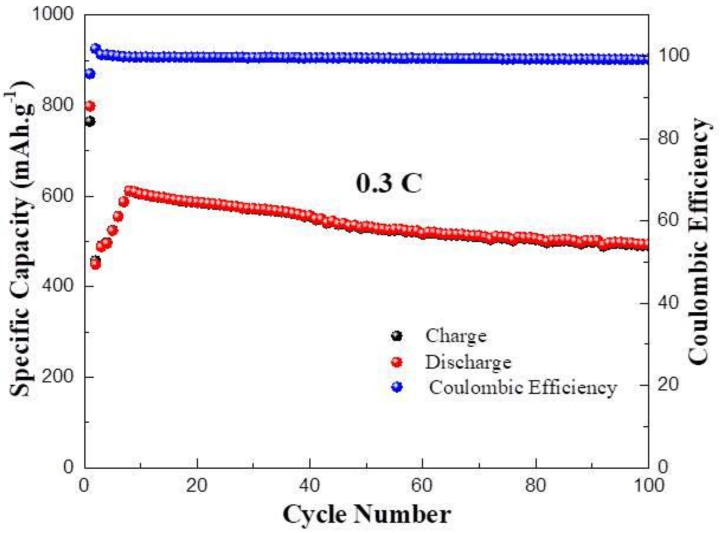

Figure S6 The cycling performance of PGBs@S at 0.3 C with a sulfur loading of 4.2 g cm^{-2}.
